# Zolpidem abuse and dependency in an elderly patient with major depressive disorder: a case report

**DOI:** 10.1186/2008-2231-22-54

**Published:** 2014-07-10

**Authors:** Maryam Pourshams, Seyed Kazem Malakouti

**Affiliations:** 1 Mental Health Research Centre, Tehran Institute of Psychiatry- Faculty of Behavioral Sciences and Mental Health, Iran University of Medical Sciences, Tehran, Iran

**Keywords:** Zolpidem, Elderly patient, Abuse, Dependency

## Abstract

**Objective:**

Zolpidem is a non-benzodiazepine hypnotic drug for treatment of insomnia. It has been introduced as a lower potential agent for dependency and abusive effects.

**Case summary:**

In this study, the reported case was a 62 years old female patient suffering simultaneously with Major Depressive Disorder and Opium Dependency. After abrupt discontinuation of zolpidem, 570 mg per day, she exhibited severe withdrawal symptoms, led her to be admitted to emergency department.

**Conclusions:**

Zolpidem has a potency to be abused with high risk of dependency and withdrawal syndromes particularly among elderly patients with comorbid anxiety/depressive symptoms/disorders.

## Background

A new generation of nonbenzodiazepine hypnotics called “Zdrugs” was introduced to clinical practice in 1980s. These drugs have fast onset of hypnotic effect and short acting duration [1].

Zolpidem (Ambien) is an imidazopyridin which is considered as nonbenzodiazepine regarding having no diazepine binding activity. However, in therapeutic dosage it affects type 1 benzodiazepine receptors selectively [2].

Zolpidem is one of the most prescribed medications to treat insomnia in elderly patients. Although some cases of abuse and dependency on zolpidem have been reported in the last decades, few cases have been identified in elderly patients with the same problem [3].

In a literature review carried out by us, only 4 reports of zolpidem dependency and withdrawal symptoms have been found in elderly patients. A summary of these reports were presented in Table 1[3-6].

**Table 1:** Studies reviewed for dependency and withdrawal syndromes of zolpidem in elderly patients

**No.**	**Authors**	**Year of publication**	**Patient’s age**	**Gender**	**Daily dose (mg)**	**Period (month)**^**+**^	**Psychiatric diagnosis**^**++**^
1	Fernandez et al.	2013	72	Female	300	Over 8	Not mentioned
2	Styliani S et al.	2009	78	Male	300	Not mentioned	Not mentioned
3	Leslie N et al.	2001	67	Female	100	18	Drug abuse^+^ symptoms of depression and anxiety
4	Liappas I.A et al.	2001	80	Female	100	Not mentioned	Without past psychiatric history

^**+**^Period (month): duration of zolpidem abuse/dependency before hospitalization.

^**++**^Psychiatric diagnosis: included past or current psychiatric diagnosis as comorbid disorder with zolpidem abuse/dependency.

This report presents a complicated 62 years female patient with dependency and withdrawal symptoms after abrupt discontinuation of 570 mg/daily of zolpidem.

## Case presentation

The case was a female patient, aged 62, married, illiterate and housewife who were admitted to Emergency Department (ED) of Rasoul Akram hospital in the June of 2013. After abrupt discontinuation of zolpidem, she presented to ED with restlessness; sever agitation, crying, severe anxiety, impatience, loss of energy, insomnia, irritability, verbal aggression, distraction, increased appetite, physical symptoms such as headaches and lightheadedness, severe shivering, and increased craving for higher dosage of opium. Urging and pressure of her close relatives was the main reason to quit the zolpidem at once. Further evaluation revealed that she had been suffering from Major Depressive Disorder (MDD) since 5 years ago that had never been noticed and managed psychiatrically. Approximately since two years before this hospitalization, the patient had started taking 10 mg of zolpidem prescribed by a general physician for her insomnia. After several months of consumption, she herself titrated of dosage for her resistant insomnia up to 570 mg per day in divided dosage. According to her report, in addition of her insomnia, depressive and anxiety comorbid symptoms were improved by every step up the zolpidem dosage.

She used to take opium for one year and quitted for the next four years. However, to alleviate her discomfort from psychological symptoms, she had started to take it orally again during the last year.

As explained by the patient, opium and zolpidem had been consumed simultaneously for its relaxing and euphoric effects.

Although the patient had a history of MDD, nevertheless, the above mentioned symptoms were beyond the criterion which could be attributed to recurrent of MDD diagnosis, according to diagnostic criteria. The onset of symptoms in one month of quitting abusive substance, make the “withdrawal” diagnosis eligible. Thus the patient was formulated with diagnosis of: 1.Zolpidem withdrawal syndrome, 2. MDD, 3. Opium dependency according to Diagnostic and Statistical Manual of Mental Disorders 4th edition- Text Revision (DSM- IV-TR).

Complete Blood Count (CBC), biochemistry test, thyroid test, Magnetic Resonance Imaging (MRI) as well as electrocardiography, echocardiography and urine test were in normal range. Urine screening test was positive for opium.

### Treatment

The patient has been managed by 1) Gabapentin (900 mg in divided doses per day), sertraline (200 mg per day) and trazodone (100 mg at bedtime). Although both of them will increase the serotonin level, however it helped to overcome the insomnia of the patient without any side effect of serotonergic syndrome. 2) Patient’s oral consumption of opium was stopped and replaced by methadone, the dosage of the methadone gradually tapered up to 40 mg per day. Close monitoring did not show any cardiac side effect of methadone and trazodone combination. After three weeks of hospitalization, she has been discharged from hospital in good condition. She was on the same medication two months after discharge and her mental and physical conditions were stable without any craving for both opium and zolpidem.

## Conclusion

Depressive disorder and insomnia is one the most prevalent mental disorders among the elderly [7]. A few studies suggested that the short-term use of benzodiazepine receptor agonists may be safe in old population [8,9]. However, sedative hypnotics are considered as potentially high-risk drugs in the elderly (aged ≥ 65 years) by the AGS 2012 Beers Criteria Update Expert Panel [10]. Recent publications indicated that benzodiazepine receptor agonists increase the risk of cognitive impairment, delirium, falling and motor vehicle accident [10]. A meta-analysis showed that an unpleasant event is more than twice as likely as improved quality of sleep [11].

Being concerned of dependency to benzodiazepine and its detrimental side effects such as forgetfulness in elderly, may encourage the general physicians prescribe zolpidem frequently without enough Psychoeducation of clients and on the other hand, the patients may take and overdose zolpidem for insomnia on their own [12].

Zolpidem is a nonbenzodiazepine hypnotic with a short half-life of 1.5 to 3.2 hours [1]. Due to its selective effect on type 1 benzodiazepine receptors, it has hypnotic and anxiolytic action without muscle relaxant and anticonvulsant effect. Lack of effects on benzodiazepine-3 receptors, it appears to result in a lower incidence of withdrawal and rebound symptoms, despite the fact that these may occur at higher doses particularly with long-term prescription [2].

Zolpidem acts in therapeutic doses selectively on receptors with α sub-unit. In contrast to benzodiazepines; it has no effect on other sub-units of such as sub-unit in amygdale whose activity by benzodiazepines has anti-anxiety effects [4]. Zolpidem acts in less selective manner in doses higher than therapeutic doses which can be of greater anxiolytic effect by affecting receptors with α_2_ sub-units. As the elderly are more likely to show mixed signs of anxiety and depression, the antianxiety effect of the medicine increases the risk of its overdose in the elderly and becomes more noteworthy when insomnia is concurrent with psychiatric disorders. Patients with psychiatric disorders often exhibit symptoms of insomnia and thus a great tendency toward abuse of alcohol, drugs, and hypnotics as their coping strategy or self-medication behavior [2,13]. It has been shown in clinical practice that although zolpidem is a safe drug, there is some risk of abuse and dependency usually related to long–term use of high doses especially in patients with previous history of substance abuse or psychiatric disorders such as depression and insomnia which can bring about the abuse and dependency on this medicine [3]. Self-escalation of the zolpidem dose can result in tolerance to the hypnotic and sedative effects of zolpidem. Low renal clearance in elderly may increase the side effect particularly in patient with taking high doses of zolpidem [14]. The elderly people seem to have a higher risk for zolpidem abuse or dependency, not only for its high frequency of sleep and mood disorders, but also for the reduction of the zolpidem clearance. In elderly population, peak plasma concentrations are 50% higher, which means that in elderly subjects even with lower doses of zolpidem, the phenomena of abuse or dependence could be envisaged [3].

Symptoms of sweating, tachycardia, tachypnea, tremors, and severe anxiety have been reported upon discontinuation of zolpidem. Discontinuation syndrome symptoms also include fatigue, nausea, flushing, panic attacks, abdominal discomfort, uncontrolled crying, emesis, delirium, and even seizure, some of which were observed in our case study [15].

The recommended dose is 5 mg at night in male and female elderly patients [10]. It is advised that secondary causes of insomnia such as psychiatric and physical conditions should be treated in elderly patients and more attention should be paid to nonpharmacological treatments such as improvement of sleep hygiene [16,17].

Although it is generally believed that zolpidem is a safe medication to use for insomnia; however it could have deleterious side effect for the elderly particularly with depressive disorder and history of substance abuse.

## Consent

Written informed consent was obtained from the patient for the publication of this report.

## Abbreviations

AGS: American Geriatrics Society; CBC: Complete blood count; DSM- IV-TR: Diagnostic and statistical manual of mental disorders 4th edition- text revision; ED: Emergency department; MDD: Major depression disorder; MRI: Magnetic resonance imaging.

## Competing interests

The authors declare that they have no competing interests.

## Authors’ contributions

MP was managing the case, drafting the paper. SKM was managing the case and scientific editing of the manuscript. Both authors read and approved the final manuscript.
